# Cyclic Loading of Jammed Granular Systems

**DOI:** 10.3390/ma15144978

**Published:** 2022-07-17

**Authors:** Piotr Bartkowski, Marta Ciemiorek, Hubert Bukowiecki, Robert Zalewski

**Affiliations:** 1 Faculty of Automotive and Construction Machinery Engineering, Warsaw University of Technology, 00-661 Warsaw, Poland; 01140749@pw.edu.pl (H.B.); robert.zalewski@pw.edu.pl (R.Z.); 2 Faculty of Materials Science and Engineering, Warsaw University of Technology, 00-661 Warsaw, Poland; marta.ciemiorek@pw.edu.pl

**Keywords:** vacuum-packed particles, granular jamming, smart materials, cyclic loading

## Abstract

This article describes the cyclic loading of jammed granular systems represented by vacuum-packed particles in compression and tension, focusing on the influence of the properties of the granular material on the mechanical response. A jammed granular system is represented by a cylindrical sample filled with polymer granules (vacuum-packed particles) and is examined in symmetric cyclic compression and tension for up to 2000 cycles and at selected values of underpressure, i.e., 0.01, 0.04 and 0.07 MPa. Force and displacement are analyzed during the test, as well as changes in granule morphology by means of microscopic observations. The conducted tests indicate that it is possible to acquire repetitive results of maximum forces in the analyzed loading rage with the condition that granules do not plasticize during loading, i.e., they are resistant to damage during loading.

## 1. Introduction

Smart materials have been studied by researchers and engineers for many years. Some of them, like magneteorological fluids (MRF) [1], electroreological fluids [2] or shape memory alloys (SMA) [3] are thoroughly tested and well commercialized. Others, like electroactive polymers (EAPs) [4] or low-melting-point materials (LMPM) [5], still require extensive research. Despite having many advantages, they all also have limitations, which reduce their application potential. The main drawbacks are their price (MRF, EAPs), low energy efficiency (LMPM) [6] and complex power and control systems [7].

A new alternative structure that can partially eliminate the problems mentioned is vacuum-packed particles (VPP) [8,9]. These are a composite that consists of polymer granulates inside a plastomer coating, whose property change is realized by granular jamming phenomena [10]. The control stimulus is usually the vacuum [11], but it can also be positive pressure [6]. This composite is extremely cheap, up to 1000 times less expensive than MR fluids, and easy to control [12], as only a simple vacuum pump is needed, which reduces the price and increases the safety of use. The mentioned advantages have been noticed in many industry areas, including medicine, automotive or robotics. The most widespread applications are ortheses, endoscopes or vacuum mattresses [13,14].

The potential of VPP structures has been recently seen by the rapidly developing area of soft robotics [15]. Such applications of those structures as universal grippers [16,17,18] or joints characterized by variable stiffness [19] have been shown. Besides being a suitable structure for grippers or manipulators, VPP are also attractive for vibration damping or crash energy absorbing [20].

The design of the above-mentioned solutions had to be preceded by a whole range of basic research that could allow us to get to know the basic mechanical properties of VPPs. Some aspects, like the influence of vacuum pressure on the mechanical properties under uniaxial tension-compression or 3-point bending, have been described in detail in [9,12], respectively. Research that shows the change of mechanical properties under high strain rate and velocity are shown in the work [8]. The damping properties of a granular jamming mechanism were tested and described in the paper [21].

A lack of research on mechanical property changes under cyclic loading is observed, which is undoubtedly an important aspect in applications such as soft manipulators or dampers. Therefore, this work is aimed at filling this gap by conducting experimental tests of VPPs. This aim is realized by conducting compression and tensile tests for up to 2000 cycles and under three selected underpressures of 0.01 MPa, 0.04 MPa and 0.07 MPa. To indicate factors responsible for property changes, granulates are analyzed by means of microscopic observations and microhardness testing.

## 2. Material and Methods

**Figure 1:**
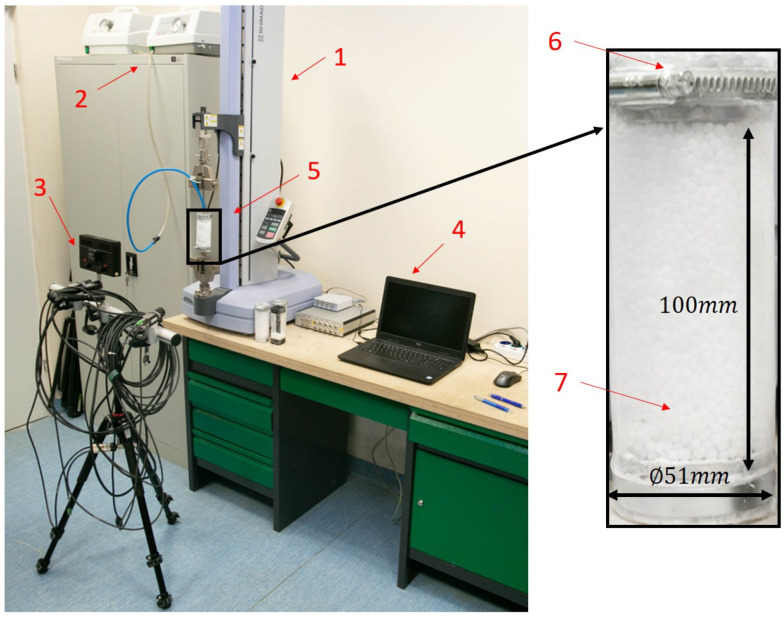
Test stand. 1—the testing machine, a SHIMADZU EZ-LX, 2—vacuum pump, 3—DANTEC DIC system, 4—main computer, 5—testing sample, 6—sample rigid disk, 7—granular core.

Cylindrical samples, presented in Figure 1, built of two rigid disks and a granular core coated by a polymer shell were tested. The sample has a diameter equal to 51 mm, a total length of 140 mm, and a granular core of 100 mm. The outer shell was made of polyethylene with a thickness of 0.15 mm and a Young’s modulus of 10 MPa. The core was filled with ball-shaped grains with a diameter of approximately 4 mm, filled with packing fractions of 57%. Three types of grains with a different parent polymer were tested, i.e., polypropylene (PP), polyoxymethylene (POM-C) and polycarbonate/acrylonitrile butadiene styrene (PC + ABS). The materials’ basic properties are listed in Table 1.

**Figure 2:**
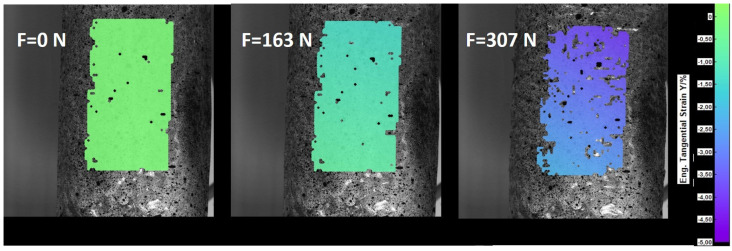
The strain distribution recorded by the DIC system for PP and vacuum pressure at 0.07 MPa.

Before each test, the cylindrical sample was placed in a dedicated mold to gain an appropriate and repetitive shape. Polymer grains were poured randomly, but with a repetitive packing fraction, which was controlled by weight. After the forming process, vacuum was applied to keep the shape, and the sample was placed in the machine yaws. Tests were performed on a testing machine, a SHIMADZU EZ-LX equipped with a DANTEC DIC system. For DIC measurements, samples were covered with a speckle pattern, which is visible in Figure 2. They are not shown in Figure 1 to visualize the granules more clearly. The speckle pattern was created using black and white spray paint. To inspect the quality, a picture was taken using a DIC set-up, and how many pixels on average cover one spot was checked, which was found to be not less than 3 × 3. The DIC DANTEC system was equipped with two 5.0 Mpx cameras and a walimex 312 LED lighting source. The working distance and off-axis angle were equal to 222.1 mm and 28.9${}^{\circ }$, respectively. Calibration was performed using the DANTEC calibration target, WD-8.00 mm-0.9×0.9×-AC1.3-0-1234. The whole test stand is shown in Figure 1. Symmetric cyclic loading tests with triangle excitation of 5 mm amplitude were performed. Tests were done for 2000 cycles and 3 values of vacuum pressure, i.e., 0.01 MPa, 0.04 MPa and 0.07 MPa. To check the the results’ repeatability, a test for each condition was done 3 times. Nine samples were prepared for each material. During the test, force, displacement and number of cycles were recorded. Additionally, to check the strain distribution, the deflection maps were obtained from the DANTEC system. DIC analysis was performed with subset size and step equal to 17 pixels and strain window equal to 3. All stresses and strains presented in the paper are engineering stress and strain calculated with respect to initial area and length. Stresses were obtained by dividing the force obtained from testing machine and initial sample area. Strains were obtained from the DIC system by averaging the area presented in Figure 2.

To evaluate changes in granule morphology during cyclic loading, specimens were taken from samples subjected to cycling loading after 200 and 2000 cycles. Granules in as-received condition were also characterized as a reference material. Granules were evaluated by light microscopy as well as by SEM, which was conducted on a Hitachi Su-70 microscope. Granules were also subjected to microhardness testing in a Vickers scale and a load of 10 g, marked as HV0.01.

## 3. Results and Discussion

Figure 2 shows the strain distribution for the testing machine’s jaw displacement of 0 mm, 1 mm and 4 mm. It can be seen that sample in uni-axial compression is deformed uniformly during the whole loading process, which proves that the assumption about engineering deformations adopted in the further part of the work is flawed with a small error.

**Figure 3:**
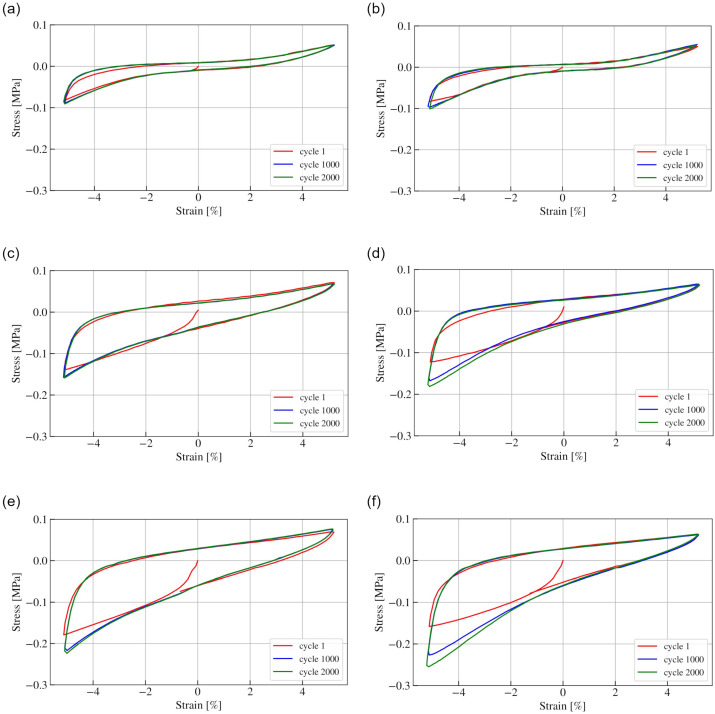
Hysteresis loops representing 1, 1000 and 2000 cycles for POM (**a**,**c**,**e**) and PP (**b**,**d**,**f**) for underpressures of 0.01, 0.04 and 0.07 MPa, respectively.

Figure 3 shows hysteresis loops of POM and PP for 1, 1000 and 2000 cycles for the vacuum pressures investigated. Hysteresis loops for POM and PC + ABS were very similar; therefore, for clarity, graphs representing only POM are presented. It can be seen that the characteristics are strongly nonlinear, and the maximum force is higher in compression than in tension, which is in line with previous research [22,23]. For each presented condition, the first loop of loading presents a different shape than after 1000 and 2000 cycles. Additionally, the general shape of the loops is similar for underpressures of 0.04 and 0.07 MPa and different for 0.01 MPa, regardless of the granules’ material. What can also be observed is that for PP granules, differences in maximum values of compression show larger differences than for PP, especially for 0.04 and 0.07 MPa of underpressure.

**Figure 4:**
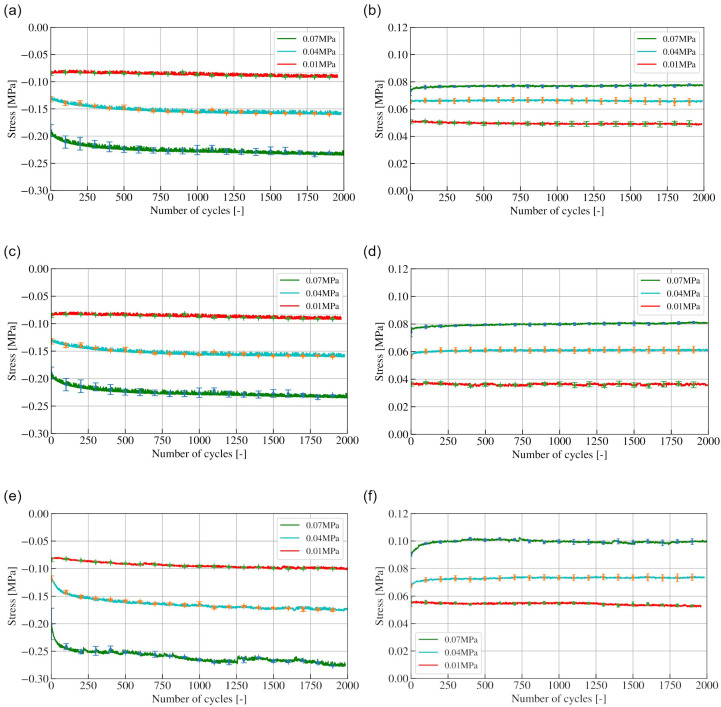
Maximum stress values as a function of number of cycles for POM compression (**a**) and tension (**b**), PC+ABS compression (**c**) and tension (**d**) and PP compression (**e**) and tension (**f**).

Maximum stresses in compression are shown in Figure 4a,c,e for POM-C, PC + ABS and PP, respectively. During compression, for POM and PC+ABS, curves representing underpressures of 0.07 MPa and 0.04 MPa are similar, as at the beginning of cyclic loading, the highest stress is reached and a sharp decrease is noted until 125 cycles are reached. A gradual stress decrease is observed up to 250 cycles, after which an almost linear dependence of stress on the number of cycles is recorded for POM and PC + ABS. As granules are poured into the cavity randomly, during the initial cycles, they move into stable positions. This explains the increasing stress value during first 125 cycles, while as more granules move into more stable positions, increasing force is needed to displace granules which have not yet reached stability. The larger the underpressure, the larger the drop, as a lower underpressure exerts a larger force between granules. After reaching final positions, after 250 cycles, force remains stable as granules are no longer displaced. For 0.01 MPa underpressure, a different trend is observed for POM-C and PC + ABS, as stress value decrease gradually, with no sharp drop at the beginning of loading. As a reduced force is exerted on granules due to the underpressure’s value, less force is needed to displace them, and the process happens gradually, over more cycles.

Curves representing the behaviour in compression of PP granules at 0.01 MPa show the same tendency as for other tested materials; however ones representing the tests at 0.04 and 0.07 MPa underpressure show a gradual decrease in maximum stress, which is in contrast with specimens filled with granules made of POM-C and PC + ABS. The POM-C and PC + ABS polymers are characterized by similar mechanical properties, i.e., their elasticity modulus, which is 2.8 and 2.5 GPa, respectively, as well as their microhardness (see Table 1).

Further differences between the behaviour of specimens filled with POM and PC + ABS granules and with PP are observed in tension, the graphical representation of which is shown in Figure 4b,d,f for POM-C, PC+ABS and PP, respectively. For 0.07 MPa underpressure, maximum stresses in tension recorded for POM and PC + ABS are between 0.04 and 0.08 MPa, depending on the underpressure value, while for PP, they are between 0.05 and 0.1 MPa.

**Figure 5:**
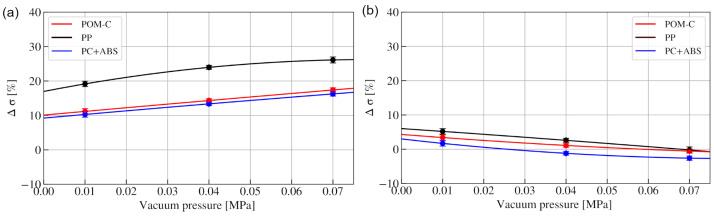
Increase in stress in compression (**a**) and tension (**b**) expressed as percentage in a function of vacuum pressure.

The percentage change (related to the initial value) in function of vacuum pressure for each type of material for compression and tension is shown in Figure 5a,b, respectively. The observed values are significantly lower for tension than for compression, and there is no significant difference observed between the tested materials. Maximum Δσ values in compression for POM-C and PC + ABS under 0.07 MPa underpressure, corresponding to 17 percent. Additionally, delta sigma in a function of vacuum pressure shows a linear dependence. For PP granules, different behaviour is observed, as higher values are reached, up to 25 percent, and Δσ values are significantly larger.

**Figure 6:**
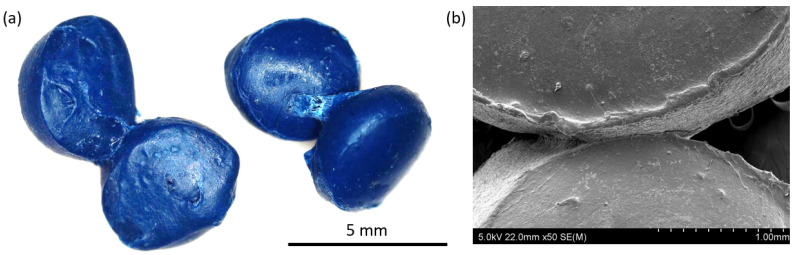
PP granules after compression testing for 250 cycles (**a**). SEM micrograph of adhered granules (**b**).

**Figure 7:**
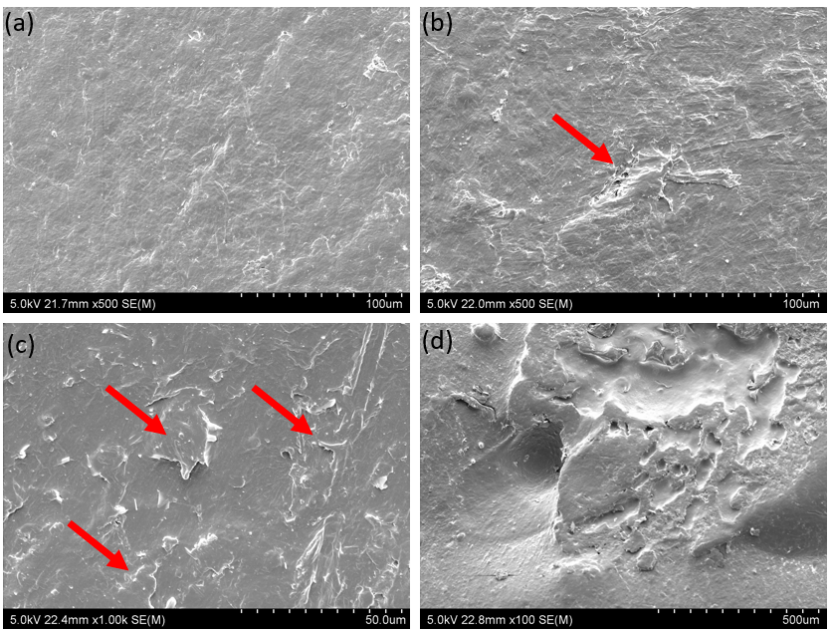
SEM micrographs of the surface of a PP granule in as-received condition (**a**), smeared material on the surface of a granule after 500 cycles in compression (**b**), protruding pieces of material form a granule after 1000 cycles in compression (**c**), and area after separation from an adhered granule (**d**).

**Figure 8:**
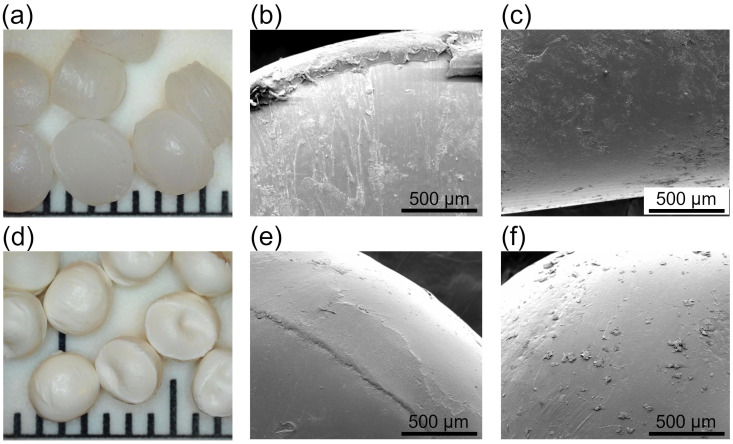
Granules of POM (**a**) and PC+ABS (**d**). One division on the scale equals 1 mm. SEM micrographs of a granule’s surface in the as-received condition for POM (**b**) and PC+ABS (**e**). Surface of granule after 2000 cycles in compression for POM (**c**) and PC + ABS (**f**).

An explanation for such differences can be found by investigating the granules’ morphology. It was observed that during cyclic loading, PP granules adhere to one another, which is shown in Figure 6a,b. This is also confirmed by the fact that the curve representing maximum stresses for compression at 0.07 MPa underpressure shows a rough uneven course, indicating fluctuations of stress cause by the adhering and breaking of the bond. Therefore, with increasing numbers of cycles, more granules are joined, and higher force is needed to separate or displace them. Various examples of this process can be found while investigating the surface of the granules before and after cyclic loading. Figure 7a presents the smooth surface of a PP granule before any loading, while Figure 7b,c shows a granule after loading cycles. In Figure 7b, the red arrow indicates visible smeared material on the surface, which was observed after 500 cycles, or material protruding from the surface of granule in Figure 7c. Figure 7d depicts what seems to be a spot where two granules were adhered and fractured. As was proven, PP granules tend to stick together during cyclic loading, which can be explained on the basis of low hardness. Its elasticity modulus is 1.6 GPa, and its microhardness is 7 HV0.01. Such features were not observed for POM and PC + ABS granules, which are shown in Figure 8a,d. Figure 8b,e presents a particle surface before loading, and Figure 8c,f presents a particle after 2000 cycles in compression, for POM and PC+ABS, respectively. The only visible change is the debris accumulated on the surface of a particle.

## 4. Conclusions

The article presents an analysis of cyclic loading of jammed granular systems under compression and tension. Test results are repeatable as evidenced by small error bars. Our study shows that the properties of the granule material are an important factor in terms of providing repetitive results of the system. The conducted research indicates that in order to have a repetitive and steady stress in compression and tension throughout 2000 cycles and the tested range of underpressures, the granules used for filling the system need to be rigid. Too soft a granule will cause unwanted phenomena to occur, such as adhering of granules, which leads to nonlinear stresses. Additionally, it was proven that the granule material has an influence on the mechanical results in compression rather than tension.

## Figures and Tables

**Table 1:** Granules’ properties.

	Density (g/cm${}^{3}$)	Mean Particle Diameter (mm)	Elasticity Modulus (GPa)	Initial Micro-Hardness HV0.01
PP	0.91	4.5	1.6	7
POM-C	1.41	4.6	2.8	22
PC + ABS	1.14	3.8	2.5	16

## Data Availability

Not applicable.
